# Sensory Loss in China: Prevalence, Use of Aids, and Impacts on Social Participation

**DOI:** 10.3389/fpubh.2019.00005

**Published:** 2019-01-24

**Authors:** Chyrisse Heine, Colette J. Browning, Cathy Honge Gong

**Affiliations:** ^1^Department of Community and Clinical Allied Health, La Trobe University, Bundoora, VIC, Australia; ^2^International Institute for Primary Health Care Research, Shenzhen, China; ^3^Centre for Research on Ageing, Health and Wellbeing, Research School of Population Health, College of Health and Medicine, The Australian National University, Canberra, ACT, Australia; ^4^ARC Centre of Excellence in Population Ageing Research (CEPAR), Sydney, NSW, Australia

**Keywords:** sensory loss, prevalence, unmet needs, social participation, China, The China Health and Retirement Longitudinal Study

## Abstract

The number of older adults with vision and/or hearing loss is growing world-wide, including in China, whose population is aging rapidly. Sensory loss impacts on older people's ability to participate in their communities and their quality of life. This study investigates the prevalence of vision loss, hearing loss, and dual sensory loss (combined vision and hearing loss) in an older adult Chinese population and describes the relationships between these sensory losses and demographic factors, use of glasses and hearing aids, unmet needs, and impacts on social participation. The China Health and Retirement Longitudinal Study is a population-based longitudinal survey conducted since 2011. The 2013 dataset for people aged 60 and over was used in this study. Items analyzed included demographic data (age, gender, education, rurality, and SES), self-reported ratings of vision (including legally blind, excellent-poor long, and short distance vision and the use and frequency of wearing glasses), hearing (excellent-poor hearing and the use of hearing aids), dual sensory loss (both poor/fair vision and hearing), and social participation. Of the sample, 80.2% reported poor/fair vision, 64.9% reported poor/fair hearing, and 57.2% had poor/fair vision and hearing. Few respondents (10%) wore glasses regularly and 20.1% wore glasses from time to time. Only 0.8% of respondents wore hearing aids although the proportion with hearing loss was high (64.9%). The proportion of unmet needs for glasses and hearing aids was 54.9 and 63.9%, respectively. Low socio-economic status (SES), poor education, and rurality were significantly associated with the prevalence of poor/fair vision and hearing, the use of glasses and hearing aids and the unmet needs of glasses/hearing aids. Poor/fair vision and/or hearing, and the unmet needs for glasses/hearing aids were significantly and negatively associated with social participation. Sensory loss is a significant health issue for older Chinese people that impacts on their social participation. Training primary care health professionals in identification and rehabilitation approaches is needed as well as increasing the numbers of vision and hearing specialists working in the field. Providing information on sensory loss and the use of aids to older adults will also help improve older adult's quality of life.

## Introduction

As adults age, they experience a range of physical changes, including changes in vision and hearing acuity. According to the WHO (1), 253 million people worldwide have vision impairment with 36 million people classified as legally blind. China accounts for ~18% of the world's blindness (2). Eighty one percent of the global population with vision impairment are aged 50 years and over with this percentage set to increase as the numbers of older people increases worldwide. The WHO (3) estimated that in 2012 there was a high proportion of people worldwide with disabling hearing loss (360 million people or 5.3% of the world's population), although disabling hearing loss in East Asia is estimated to be much higher at 22% (2). Of the proportion of people worldwide with hearing loss, approximately one-third are older adults aged 65 years and over. The burden of disabling hearing impairment is highest for older adults in Southern Asia and the Asia pacific region (4).

In China, few population–based studies have explored the prevalence and impact of the co-occurrence of vision and hearing loss, known as Dual Sensory Loss (DSL). Dual Sensory Loss (DSL) is defined as “the acquired loss, in various degrees of severity of both vision and hearing acuity, associated with aging and prevalent in older adults” (5) (p. 1). The focus of the current paper is to investigate the prevalence and impact of vision, hearing and dual sensory loss in a representative sample of older Chinese.

Population-based studies of vision loss in China have shown the extent of the problem especially in older age groups. Zhao et al. (6) analyzed the findings of the China Nine-Province Survey of 45,747 adults aged 50 years and over living in rural China and found that the prevalence of visual impairment ranged from 3.76 to 38.40% and blindness ranged from 0.50 to 14.80%. In the 60 year and older age group, the prevalence of visual impairment was 17.05 and 3.91% for blindness. In addition, the increased prevalence of visual impairment and blindness was associated with older age, female gender, lack of education, and geographical area (or province). Zhang et al. (7) surveyed 5,770 persons aged 40 years and older in both urban and rural communities in 2013 in Baotou, China. They defined low vision as visual acuity in the better eye as 20/400-20/60, and blindness as visual acuity in the better eye as < 20/400. In this study overall bilateral prevalence rates of low vision and blindness amongst those aged 40 years and over were 3.66 and 0.99%, respectively, and the proportion of low vision increased rapidly from 2.61% for those aged 60–69 years to 28.57% for those aged 90 years and over. Wang et al. (8) evaluated survey data from the Global Burden of Diseases, Injuries, and Risk Factors study 2015 and concluded that the prevalence of eye diseases in China had increased both in prevalence and type from 1990 to 2015, and will continue to increase until 2020 due to population growth and population aging. The burden from vision loss due to eye disease was ranked 11th amongst the causes of health loss in China in 2015.

Hearing impairment is also a prevalent condition in China that increases with age. A 1998 study conducted in Shanghai, China found that out of 2,044 people aged 65–74 years, 486 people (23.8%) rated their hearing as poor, whilst out of 1,050 people aged 75 years and over, 415 people (39.5%) rated their hearing as poor (9). Similarly, in a study conducted in Sichuan, China, Liu et al. (10) used ear and hearing examinations and found that out of a sample of 11,421 people aged 60 years and over, there were 1,465 cases (35.18%) of people with a hearing loss. Of these older adults aged 60 years and over, the prevalence rate of hearing loss was 12.83%. In a more recent study, Gong et al. (11) investigated the prevalence of self-reported hearing loss in 6,984 older adults aged 60 years and older in China. Findings of this study suggested that the prevalence of hearing loss was 58.85% with age and gender highly correlated with hearing loss. In 65–69 year olds, the prevalence of hearing loss was 57.59% which increased to 81.36% in adults aged 80 years and over. Hearing loss was also more prevalent in males (60.87%) than females (56.99%) and there were significant variations in hearing loss by urban/rural residence, education, and annual HH income.

Prevalence estimates of DSL (5) vary greatly between studies due to for example, differences in sample size or sample characteristics (12). In a recent study by Swenor et al. (13) the prevalence of DSL (or Dual Sensory Impairment) in a US sample was estimated as 11.3% of all adults aged 80 years or over. This is in contrast to Caban et al. (14) who investigated 1,110 community residing people in the US and found the prevalence of DSL to be 7.3% in participants aged 69–79 years and 16.6% in participants aged 80 years and over.

Limited prevalence data is available on DSL in the Chinese population. In a study of 2,003 older residents (aged 60 years and over) in Hong Kong, Chou and Chi (15) found that 20.0% had poor vision, 17.5% had hearing impairment, and 6.5% had vision and hearing loss. The prevalence of dual sensory loss is expected to increase with the aging of the population (16) and particularly in China, with increases in the number of older people aged 80 years and over.

Sensory loss impacts on older peoples' abilities to communicate and participate in society and can contribute to decreased productivity and quality of life (17, 18).

Communication difficulty is significantly impacted by sensory loss and consequently often leads to decreased social interaction and participation. In particular, people with vision loss have difficulty perceiving visual cues such as visual detail, distance perception, illumination and facial acuity of the communication partner (19). People with severe visual loss (low vision or legal blindness) thus frequently experience difficulty seeing their communication partner's face and therefore have difficulty with lipreading or perceiving non-verbal cues such as gesture, facial expression, and body posture. This decreased visual perception interferes with effective verbal communication, particularly in social situations. People with severe vision loss usually rely on the auditory modality to compensate for their difficulties. However, when auditory acuity is also reduced and cannot compensate for diminished visual acuity (as occurs in DSL), effective communication is compromised (17).

Visual aids are not always helpful for people with severe vision loss and many older adults with hearing loss or DSL do not successfully use amplification or other assistive listening devices successfully to improve their hearing (20). In China, many adults with vision and hearing loss do not use aids to assist with their sensory loss. Although self-reported vision problems are common in China, particularly rural China, there is comparatively limited use of glasses due to poverty, misinformation, and mistaken views (for example that wearing glasses leads to further loss of vision) (21, 22). Similarly, there is less uptake of hearing aid use than expected for the large number of people in China with hearing loss (23). Reasons posited by these authors for poor uptake of hearing aids include traditional attitudes toward hearing loss in older people (“it is a normal part of aging”), financial constraints, unfamiliarity with hearing aids, and difficulty manipulating hearing aids. Furthermore, these authors found that many older people find the enhanced signal too loud, feel that the signal has a muffled effect and believe that the hearing aid does not assist with speech recognition. These authors also concluded that hearing healthcare services for older people in China is still under-developed.

Sensory loss in older people in China is thus a major chronic health issue that has the potential to impact on the social participation and the quality of life of older Chinese. The availability and use of sensory aids are important components in assisting older people with sensory loss to function at their full potential.

There are few population-based studies that examine in detail vision, hearing and dual sensory loss in China. The China Health and Retirement Longitudinal Study (CHARLS) used in this study is a national representative population-based longitudinal survey of people aged 45 years and over, that includes a number of items on sensory loss and aid use as well as social participation.

The aims of this study are to:

1. estimate the prevalence of self-rated vision and hearing and DSL in the older adult cohort (60 years and over) of the CHARLS study;
2. evaluate the relationship between the prevalence and severity of self-reported sensory loss and age, gender, socioeconomic status, and rurality;
3. evaluate the relationship between the prevalence of self-reported sensory losses and the uptake of visual aids (glasses) and auditory aids (hearing aids) to assess unmet needs; and,
4. evaluate the relationship between self-reported sensory loss, visual aids (glasses) and auditory aids (hearing aids) use and social participation.

## Methods

### Data Source

The CHARLS is the first nationally representative and longitudinal survey of people aged 45 years or above on aging and health in China. It was designed by following the protocols of the Health and Retirement Survey (HRS) in the US and conducted by the China Center for Economic Research at Peking University (24). Face-to-face interviews in respondents' homes collected detailed information on demographic characteristics, social and economic conditions, self-rated health, chronic diseases, activities of daily living (ADL) limitations, disabilities, and psychological well-being amongst other variables. In the first wave of CHARLS in 2011, participants were randomly sampled using a multi-stage probability-proportional-to-size technique, stratified by regions and then by urban districts or rural counties and per capita gross domestic product (GDP).

In the first wave of CHARLS in 2011, 17,596 respondents aged 45 years and over were surveyed. In the second wave in 2013, the sample size increased to a total of 18,246 respondents, in which 14,988 were from Wave 1.

For our investigation of hearing and vision loss, which are most prevalent in older people, we only included older adults (those aged 60 years and over) in the analyses (*n* = 8,268). There are some missing data or non-response for each of our study variables. For instance, 7,401 have reported information on vision capacity, 7,396 on vision capacity for long distance, 7,398 on near vision capacity, 8,193 on wearing glasses or not, 7,430 on using hearing aid or not, and 7,431 on social activities.

Missing data have not been imputed. We used observations with responses to related variables to conduct descriptive analyses of the prevalence rates of vision/hearing loss by age, gender, and SES. The correlational and regression analyses between social participation and vision/hearing loss, were restricted to the final 7,212 respondents with reported information on all the related variables of social participation, vision/hearing capacity, and the use of glasses/hearing aids used in the final regression models.

### Methods

First the prevalence rates of self-reported vision and hearing loss and unmet needs for glasses or hearing aids by age, gender, urban/rural residence, education, socio-economic status (SES) (relative living standard and quintile of household expenditure) were calculated followed by Spearman correlations between the prevalence rates of vision and hearing status, unmet needs and social participation. Finally, a multivariate regression analysis was performed to examine the conditional associations of poor/fair vision/hearing, DSL and the unmet needs for glasses or hearing aids with social participation by controlling for age group and gender. The ordered logit model was run for the number of types of social activities, while the logit model was run for whether respondents participated in any, leisure, helping others or learning social activities. The multivariate regression models used in this study are simple without controlling for other important factors, such as disability, deafness, blindness, or psychological problems. However, the modeling approach serves the broad purpose of the paper to investigate the conditional associations between social participation, vision/hearing loss, and the use of sensory aids.

The statistical software we have used for the analysis is STATA/SE 15.1. Individual weights denoting the inverse of the probability that the observation are included because the sampling design with household and individual non-response adjustments, are used for our analysis.

### Measures

In CHARLS 2013, there are three questions asking respondents about their vision: (1) Do you usually wear glasses or corrective lenses (1 = Yes, 2 = Legally blind, 3 = No, 4 = Sometimes)? (2) How good is your vision for seeing things at a distance, like recognizing a friend from across the street (with glasses or corrective lenses if you wear them, 1 = excellent, 2 = very good, 3 = good, 4 = fair, or 5 = poor)? (3) How good is your near vision, like reading ordinary newspaper print (with glasses or corrective lenses if you wear them, 1 = excellent, 2 = very good, 3 = good, 4 = fair, or 5 = poor)? The two questions for hearing are, “Do you ever wear a hearing aid (1 = yes or 2 = no)?” “Is your hearing excellent, very good, good, fair, poor, or very poor (with a hearing aid if you normally use it and without if you normally don't)?”

In the current literature, vision/hearing loss has been defined either objectively passing certain thresholds in medical examination/testing, e.g., Kuang et al. (25) or subjectively self-reporting poor/fair vision/hearing capacities, e.g., Yu et al. (9) reflecting needs for sensory aids to help daily and social activities. In this study, for the analysis of the prevalence of DSL and its association with social participation, both vision and hearing, subjective ratings were categorized into two groups: good (including excellent/very good/good), and poor (including fair/poor).

Based on these responses, we created five different variables to measure vision or hearing loss: (1) having poor/fair vision (for either long distance or near vision); (2) having poor/fair vision for long distance; (3) having poor/fair near vision; (4) having poor/fair hearing; (5) dual sensory loss (having poor/fair vision and hearing). In addition, we included self-reported blindness.

Since wearing glasses or using hearing aids could be influenced by both affordability and services needs, a variable was derived to measure the unmet needs for glasses and for hearing aid/s use as follows: (1) Unmet needs for glasses = a vision loss was reported but glasses were not used; (2) unmet needs for hearing aid/s = if a hearing loss was reported but hearing aid/s were not used. The counterpart of unmet needs includes those without vision/hearing loss who do not need sensory aids, and those with vision/hearing loss who use glasses/hearing aid/s regularly or from time to time.

Socioeconomic status (SES) is an economic and sociological combined total measure of a person's work experience and of an individual's or family's economic and social position in relation to others, based on their income, education, and occupation (26). There are a number of measures of socio-economic status (SES) in CHARLS which might be related to DSL and the use of sensory aids, including: (1) household income; (2) household expenditure, (3) self-reported relative living standards, and (4) individual educational attainment. Estimating income for old people in China is complicated and fluctuates. Older people have multiple income sources from their wages, age pension, savings, investments, rent, intergenerational transfer and children's support amongst others In order to characterize the impact of the large variations across regions in both income and living costs, the relative living standard and quintiles of household annual expenditure were used together to measure SES (26). Household expenditure includes household spending on food and non-food (including eating out, alcohol, cigarettes, cigars, and tobacco) as well as the market value of food and other products that members of the household consumed and that they grew or produced by themselves. The weekly value of spending from the survey is timed by 52 weeks in order to obtain the yearly value. The quintile of household expenditure is calculated based on the whole household expenditure at a national level among all respondents aged 45 and over in CHARLS 2013. The relative living standard is self-reported by respondents by answering the question: “Compared to the average living standard of people in your city or county, how would you rate your standard of living relative to those in your city/county: much better, a little better, about the same, a little worse, much worse?” Answers were grouped into three categories: “better;” “worse;” and “about the same,” since using five groups did not enhance the interpretation beyond using the three categories used in the model.

Educational attainment was categorized into three groups: (1) primary or under schooling; (2) secondary schooling; (3) college or above degrees. The urban and rural areas were defined according to the most recently published statistical standard by the National Bureau of Statistics of China (NBS) (27), where urban areas include both communities and villages located within access to the city or town facilities, while rural areas include only villages out of the city or town facilities.

To measure social participation in the CHARLS 2013 dataset, respondents were asked “Have you done any of these activities in the last month (multiple options)? (1) Interacted with friends, (2) Played Ma-jong, played chess, played cards, or went to community club, (3) Provided help to family, friends, or neighbors who do not live with you, and who did not pay you for the help, (4) Went to a sport, social, or other kind of club, (5) Took part in a community-related organization, (6) Done voluntary or charity work, (7) Cared for a sick or disabled adult who does not live with you and who did not pay you for the help, (8) Attended an educational or training course, (9) Stock investment, (10) Used the Internet, (11) Other (12) None of these. Options 1, 2, 4, and 5 are defined as leisure activities, options 3, 6, and 7 as social activities helping others out and options 8, 9, 10, and 11 as learning activities for new knowledge.

Five variables were generated to define social activities based on the respondents' responses to this question and are as follows: (1) the number of types of social activities as described above; (2) any of these social activities; (3) any of the leisure activities; (4) any of the activities helping others out; and (5) any learning activities for new knowledge.

## Results

Table 1 reports the sample size, weighted, and unweighted proportions by individual characteristics of the respondents from both the full and restricted CHARLS samples. The full sample (8,268 respondents) was used for descriptive analysis (Tables 1–3), although in some of the analyses, the number of respondents is <8,268 due to missing data for some variables as noted above. The restricted sample (7,212) was used to check unconditional and conditional associations between hearing/vision loss and social activities (Tables 4–6). There were no large differences between weighted and unweighted proportions for the full and restricted samples hence no systematic bias was expected by using the full or restricted sample.

**Table 1:** Sample size, unweighted, weighted proportions by individual characteristics for full and restricted samples.

	**Full sample**			**Restricted sample**		
	**Sample size (*n*)**	**Unweighted (%)**	**Weighted (%)**	**Sample size (*n*)**	**Unweighted (%)**	**Weighted (%)**
All respondents aged 60+	8,268	100	100	7,212	100	100
(1) Male	4,093	49.5	49.1	3,587	49.7	49.6
(2) Female	4,173	50.5	50.9	3,625	50.3	50.4
(1) Aged 60–64	2,677	32.4	30.4	2,418	33.5	33.5
(2) Aged 65–69	2,197	26.6	25.3	2,020	28.0	28.0
(3) Aged 70–74	1,539	18.6	18.3	1,360	18.9	18.9
(4) Aged 75+	1,855	22.4	26.0	1,414	19.6	19.6
(1) Rural	4,957	60.0	59.9	4,395	60.9	60.6
(2) Urban	3,311	40.0	40.1	2,817	39.1	39.4
(1) Primary or under	4,768	57.7	58.5	4,093	56.8	57.2
(2) Second schooling	3,343	40.4	39.9	2,991	41.5	41.2
(3) College and above	155	1.9	1.6	128	1.8	1.6
(1) Better living standard	256	3.1	2.8	242	3.4	3.2
(2) Average living standard	1,674	20.3	19.6	1,621	22.5	22.3
(3) Worse living standard	4,151	50.2	49.5	4,030	55.9	56.1
(4) Living standard not reported	2,187	26.5	28.1	1,319	18.3	18.5
(1) Household expenditure quintile 1	2,095	26.1	27.3	1,627	22.7	22.4
(2) Household expenditure quintile 2	1,926	24.0	23.2	1,828	25.5	25.1
(3) Household expenditure quintile 3	1,558	19.4	19.0	1,466	20.4	20.4
(4) Household expenditure quintile 4	1,283	16.0	15.7	1,197	16.7	16.7
(5) Household expenditure quintile 5	1,150	14.4	14.8	1,064	14.8	15.4
(1) Good vision	1,404	19.0	19.8	1,372	19.0	19.8
(2) Poor/fair vision	5,997	81.0	80.2	5,840	81.0	80.2
(1) Good hearing	2,555	34.4	35.1	2,496	34.6	35.1
(2) Poor/fair hearing	4,875	65.6	64.9	4,716	65.4	64.9
(1) Without DSL	3,079	41.7	42.8	3,030	42.0	42.8
(2) With DSL	4,298	58.3	57.2	4,182	58.0	57.2
(1) Met needs for glasses	1,940	26.2	25.4	1,894	26.3	25.4
(2) Unmet needs for glasses	4,056	54.8	54.9	3,945	54.7	54.8
(3) Good vision no need for glasses	1,403	19.0	19.8	1,371	19.0	19.8
(1) Met needs for hearing aid	41	0.6	0.5	38	0.5	0.5
(2) Unmet needs for hearing aid	4,798	65.0	64.3	4,643	64.8	64.8
(3) Good hearing no need for aid	2,547	34.5	35.2	2,488	34.7	34.7
Social activities: reported	7,431			7,212	
(1) Social activities: any (yes)	4,040	54.4	54.7	3,928	54.5	54.9
(2) Social activities: any(no)	3,391	45.6	45.3	3,284	45.5	45.1
(1) Social activities: leisure (yes)	3,731	50.2	50.3	3,627	50.3	50.6
(2) Social activities: leisure (no)	3,700	49.7	49.6	3,585	49.7	49.4
(1) Social activities: helping others (yes)	800	10.8	10.4	772	10.7	10.5
(2) Social activities: helping others (no)	6,631	89.2	89.5	6,440	89.3	89.5
(1) Social activities: learning (yes)	276	3.3	3.7	269	3.73	3.82
(2) Social activities: learning (no)	7,155	96.64	96.22	6,943	96.27	96.18

*Data source: CHARLS 2013. http://charls.pku.edu.cn/en*.

(1) *Among all the 8,268 respondents in the full sample, all of them have reported information on age and urban/rural residence, 8,266 on gender and education, 6,081 on relative living standard, 8,012 on household expenditure, 7,401 on either long or near vision capacity, 7,396 on long vision capacity, 7,398 on near vision capacity, 7,430 on hearing capacity, 7,377 on dual sensory loss, 7,399 on unmet needs for glasses, 7,386 on unmet needs for hearing aids, and 7,431 on social activities*.

(2) *Full sample (8,268 respondents) is used for descriptive analysis, while restricted sample (7,212) is used to check associations between hearing/vision loss and social activities*.

(3) *Weighted and unweighted proportions (%) are calculated based on respondents in full or restricted samples with reported information on each of the variables. For instance, in the full sample, 8,266 have reported gender information, and the proportion of males and females are calculated based on 8,266 instead of 8,268 respondents*.

**Table 2:** Poor/fair vision and/or hearing by age groups and gender among older Chinese aged 60+, 2013.

**Weighted proportion (%)**	**Sample size CHARLS 2013**	**Poor/fair vision for long distance/near vision**	**Poor/fair vision for long distance**	**Poor/fair near vision**	**Wearing glasses regularly**	**Wearing glasses from time to time**	**No glasses**	**Poor/fair hearing**	**Wearing hearing aid/s**	**Dual poor/fair vision & hearing**	**Unmet needs for glasses**	**Unmet needs for hearing aid**
**Persons**	*n*	%	%	%	%	%	%	%	%	%	%	%
Aged 60–64	2,677	80.6	64.6	68.3	9.8	22.9	67.2	61.4	0.6	55.2	53.6	60.8
Aged 65–69	2,197	80.2	70.2	67.3	9.8	23.7	66.0	64.4	0.5	56.6	53.2	63.3
Aged 70–74	1,539	81.5	72.2	66.8	11.8	19.7	67.4	67.0	0.7	60.0	54.3	66.0
Aged 75+	1,855	78.7	70.7	62.8	9.0	13.5	76.5	68.9	1.4	58.5	59.3	67.2
Total aged 60+	8,268	80.2	68.9	66.6	10.0	20.1	69.4	64.9	0.8	57.2	54.9	63.9
**Men**	*n*	%	%	%	%	%	%	%	%	%	%	%
Aged 60–64	1,278	77.4	59.2	64.7	12.2	23.8	63.8	61.1	0.4	53.8	49.1	60.5
Aged 65–69	1,125	77.9	66.8	66.4	10.8	25.4	63.2	64.9	0.6	55.2	49.9	63.7
Aged 70–74	787	80.7	68.8	67.3	12.7	23.4	63.6	70.3	1.0	62.5	49.8	68.9
Aged 75+	903	76.0	67.7	60.5	13.1	16.4	70.3	69.1	2.4	57.5	54.4	67.1
Total aged 60+	4,093	77.9	65.0	64.7	12.2	22.3	65.2	65.7	1.1	56.7	50.6	64.5
**Women**	*n*	%	%	%	%	%	%	%	%	%	%	%
Aged 60–64	1,398	83.4	69.5	71.6	7.6	22.0	70.2	61.6	0.7	56.5	57.6	61.1
Aged 65–69	1,071	82.7	73.9	68.3	8.8	21.8	68.9	63.8	0.4	58.1	56.7	62.8
Aged 70–74	752	82.3	75.7	66.3	10.9	16.0	71.9	63.7	0.4	57.4	59.0	63.1
Aged 75+	952	81.3	73.6	65.1	5.4	11.0	82.1	68.7	0.5	59.6	64.0	67.4
Total aged 60+	4,173	82.5	72.7	68.3	7.9	17.9	73.4	64.2	0.5	57.8	59.0	63.3

*Data source: CHARLS 2013. http://charls.pku.edu.cn/en*.

(1) *The proportions (%) here are weighted proportions based on observations with reported information on each of the variables. (4) The proportions of unmet needs for glasses/hearing aid are based on the proportions of respondents with vision/hearing loss but without glasses/hearing aid among all respondents who have reported vision/hearing capacities. As shown in Table 1, its counterpart includes both respondents who do not have vision/hearing loss, and those with vision/hearing loss but with glasses/hearing aid*.

**Table 3:** Poor/fair vision and/or hearing by SES among older Chinese aged 60+, 2013.

**Weighted proportion (%)**	**Sample size CHARLS 2013**	**Poor/fair vision for long distance/near vision**	**Poor/fair near vision**	**Poor/fair near vision**	**Wearing glasses regularly**	**Wearing glasses from time to time**	**No glasses**	**Poor/fair hearing**	**Wearing hearing aid**	**Dual poor/fair vision & hearing**	**Unmet needs for glasses**	**Unmet needs for hearing aid**
By education	*n*	%	%	%	%	%	%	%	%	%	%	%
Primary or under	4,768	82.21	71.7	67.8	6.0	15.0	78.4	66.5	0.7	59.1	63.0	65.8
Second schooling	3,343	77.9	65.4	65.3	15.0	27.4	57.2	63.2	0.9	55.2	44.5	62.0
College and above	155	69.28	59.1	55.1	30.8	23.7	45.5	49.2	1.6	42.6	30.0	41.9
Total aged 60+ with educational information	8,266	80.2	68.9	66.6	10.0	20.1	69.4	64.9	0.8	57.0	54.9	63.9
By residence	*n*	%	%	%	%	%	%	%	%	%	%	%
Rural	4,957	82.2	72.0	68.0	7.3	18.0	74.2	68.1	0.6	61.0	60.0	67.4
Urban	3,311	77.21	64.0	64.0	14.1	23.0	62.2	60.0	1.1	52.1	47.5	58.4
Total aged 60+	8,268	80.2	68.9	67.0	10.0	20.1	69.4	64.9	0.8	57.2	54.9	63.9
By living standard	*n*	%	%	%	%	%	%	%	%	%	%	%
Better living standard	256	73.45	57.1	60.6	15.7	19.1	64.4	57.4	1.9	47.2	46.2	55.0
Average living standard	1,674	77.67	65.1	64.1	10.5	22.0	67.4	62.3	1.0	53.2	52.5	60.7
Worse living standard	4,151	82.21	71.4	68.5	9.3	20.3	69.9	67.1	0.5	59.8	57.7	66.2
Living standard not reported	2,187	78.5		64.6	10.2	18.3	70.3	1.2	62.9	1.2	50.7	62.1
Total aged 60+	8,268	80.2	69.2	66.6	10.0	20.1	69.4	0.6	64.9	0.8	54.87	63.88
By expenditure	*n*	%	%	%	%	%	%	%	%	%	%	%
HH expenditure quintile 1	2,095	80.68	71.5	65.2	8.6	15.6	74.6	66.0	1.1	58.9	58.1	65.0
HH expenditure quintile 2	1,926	82.33	70.8	70.1	9.9	20.0	69.9	68.0	0.8	60.3	57.8	67.1
HH expenditure quintile 3	1,558	81.79	70.1	68.7	8.0	22.1	69.4	63.2	0.6	56.2	57.1	62.1
HH expenditure quintile 4	1,283	78.39	65.3	64.1	12.7	22.2	64.9	63.6	0.5	55.2	50.0	62.9
HH expenditure quintile 5	1,150	76.17	64.7	62.7	12.0	23.6	63.8	62.3	0.7	53.5	47.7	60.6
Total aged 60+ with information on HH expenditure	8,012	80.3	69.0	66.6	10.0	20.1	69.4	64.9	0.8	57.3	54.9	63.9

*Data source: CHARLS 2013. http://charls.pku.edu.cn/en*.

(1) *The proportions (%) here are weighted proportions based on observations with reported information for each of the variables. (4) The proportions of unmet needs for glasses/hearing aid are based on the proportions of those respondents with vision/hearing loss but without glasses/hearing aid among all respondents who have reported vision/hearing capacities. As shown in Table 1, its counterpart includes those who do not have vision/hearing loss, and those with vision/hearing loss but with glasses/hearing aid*.

**Table 4:** Participation in social activities (%) by self-rated vision and hearing, and unmet needs.

**Participation (%) of social activity**	**All restricted sample**	**By vision capacity**		**By hearing capacity**		**By DSL**		**By needs for glasses**		**By needs for hearing aids**	
**By social activity type**	**Average**	**Good vision**	**Poor/fair vision**	**Good hearing**	**Poor/fair hearing**	**Without DSL**	**DSL**	**Good vision or with glasses**	**Unmet needs for glasses**	**Good hearing or with health aids**	**Unmet needs for hearing aid**
Social activity: any (yes)	54.9	58.5	54.1*	58.9	52.8*	58.1	52.5*	60.3	50.5*	58.7	52.8*
Social activity: leisure(yes)	50.6	54.0	49.8*	53.6	48.9*	53.3	48.6*	55.0	47.0*	53.5	48.9*
Social activity: helping (yes)	10.5	11.9	10.2	11.8	9.8*	11.4	9.9*	12.6	8.8*	11.8	9.8*
Social activity: learning(yes)	3.8	5.4	3.4*	5.5	2.9*	5.2	2.8*	5.6	2.3*	5.5	2.9*

The numbers reported here are the weighted proportions (%) of respondents who have reported participation in any social activities. These numbers are calculated based on the restricted sample (7,212 respondents) used in the final regression models. The differences in this table are all statistically significant at a significant level 5% (as indicated by *), except for the insignificant correlation between poor/fair vision and social activity of helping others. The reference groups for the comparisons of differences in proportions are: good vision, good hearing, without DSL, good vision or with glasses, good hearing or with health aids. Data source: CHARLS 2013. http://charls.pku.edu.cn/en.

**Table 5:** Spearman correlation between social activity participation and poor/fair vision and/or hearing, and unmet needs.

**Spearman correlations**	**Social activities: number of types**	**Social activities: any (yes/no)**	**Social activities: leisure (yes/no)**	**Social activities: helping others (yes/no)**	**Social activities: learning (yes/no)**
Poor/fair vision	−0.044*	−0.028*	−0.028*	−0.018	−0.046*
Poor/fair hearing	−0.054*	−0.042*	−0.035*	−0.036*	−0.046*
DSL	−0.062*	−0.047*	−0.046*	−0.025*	−0.049*
Unmet needs for glasses	−0.125*	−0.101*	−0.088*	−0.068*	−0.080*
Unmet needs for hearing aid	−0.053*	−0.040*	−0.034*	−0.034*	−0.049*

(1) The spearman correlations are based on the restricted sample (7,212 respondents) used for the final regression models;

(2) * indicates statistically significant at a significant level of 5%. Only the correlation between social activities of helping others and poor/fair vision is insignificant. Data source: CHARLS 2013. http://charls.pku.edu.cn/en.

**Table 6:** Estimated results from multivariate regression models on sensory loss and social activities.

**All persons aged 60+**	**Social activities: number of types**			**Social activities: any (yes/no)**			**Social activities: leisure (yes/no)**			**Social activities: helping others (yes/no)**			**Social activities: learning (yes/no)**		
	**Coefficient**	**Std. err**.	***P*-value**	**Coefficient**	**Std. err**.	***P-*value**	**Coefficient**	**Std. err**.	***P-*value**	**Coefficient**	**Std. err**.	***P-*value**	**Coefficient**	**Std. err**.	***P-*value**
**60–64 (REFERENCE)**															
65–69	−0.022	0.071	0.760	0.059	0.072	0.411	0.000	0.076	0.998	−0.109	0.100	0.276	0.195	0.252	0.439
70–74	−0.142*	0.070	0.042	0.001	0.078	0.994	−0.002	0.078	0.978	−0.328*	0.125	0.009	−0.120	0.197	0.544
75+	−0.304*	0.079	<0.001	−0.159	0.083	0.054	−0.092	0.082	0.262	−1.164*	0.156	<0.001	−0.305	0.214	0.155
**MALE (REFERENCE)**															
Female	−0.115*	0.055	0.035	−0.051	0.058	0.375	0.001	0.059	0.992	−0.098	0.086	0.258	−0.265	0.176	0.133
Poor/fair vision	0.148	0.112	0.186	0.190	0.119	0.111	0.135	0.126	0.284	0.002	0.155	0.990	0.330	0.371	0.373
Poor/fair hearing	−0.247	0.297	0.407	−0.314	0.302	0.300	−0.159	0.301	0.597	−0.310	0.506	0.541	0.281	0.528	0.595
DSL	−0.005	0.160	0.977	0.016	0.166	0.923	−0.027	0.167	0.871	0.249	0.232	0.284	−0.110	0.388	0.778
Unmet needs for glasses	−0.454*	0.061	<0.001	−0.444	0.068	<0.001	−0.351	0.071	<0.001	−0.384*	0.098	<0.001	−0.925*	0.231	<0.001
Unmet needs for hearing aid	−0.003	0.270	0.992	0.076	0.272	0.781	0.016	0.270	0.952	−0.032	0.489	0.948	−0.830	0.509	0.103
Intercept				0.482	0.098	<0.001	0.236	0.099	0.016	−1.551	0.126	<0.001	−2.567	0.206	<0.001
Sample size	7,212			7,212			7,212			7,212			7,212	
R2	0.010			0.0105			0.006			0.027			0.039	

* *indicates statistically significant at a significant level 5%*.

*Data source: CHARLS 2013. http://charls.pku.edu.cn/en*.

In the 2013 CHARLS sample, there were 8,268 respondents aged 60 years and over, of which, 49.5% were men whilst 50.5% were women. More specifically, there were 32.4% of respondents aged 60–64 years, 26.6% of respondents aged 64–69 years, 18.6% of respondents aged 70–74 years, and 22.4% of respondents aged 75 years and over. Sixty percent of respondents were from rural areas whilst 40.0% were from urban areas.

The educational attainment was very low in this sample. Of all the respondents aged 60 years and over, only 1.9% had a college or above degree, 40.4% had secondary schooling, and 57.7% had primary or under primary schooling. About 3.1% of all respondents reported having a better living standard than the average in their cities or counties, 20.3% reported about the same and 50.2% reported a worse living standard than the average in their cities or counties. About 26.1% of respondents were located in the lowest quintile of household expenditure, while 14.4% were in the highest quintile of household expenditure (based on all respondents aged 45 and over in CHARLS 2013).

Amongst all respondents aged 60 years and over, the following information was evident: (1) 81.0% reported poor or fair vision capacity for either long or near distance, <1% of respondents across the age groups reported blindness, 65.6% reported poor or fair hearing capacity, and 58.3% reported Dual Sensory Loss (DSL); (2) 19.0% of respondents reported good vision with no need for glasses, 26.2% wore glasses and 54.82% reported having no glasses even though they reported poor or fair vision; (3) 34.5% of respondents reported good hearing without the need for hearing aids, very few respondents (0.6%) used hearing aids and 65.0% of respondents reported having a hearing loss, yet did not use hearing aids.

Of the 7,431 respondents (89.9% of the full sample) who reported information about their social activities, 54.4% of respondents reported having any social activities in last month, 50.2% reported participating in leisure activities, 10.8% reported helping others and 3.3% participated in learning activities.

### Prevalence of Sensory Loss and Aid Use: Age and Gender Effects

The prevalence rates of self-reported vision loss, hearing loss and DSL, the use of aids (glasses and hearing aid/s), and social participation were obtained and analyzed by age, gender, education, urban/rural residence, and SES (relative living standard, quintile of household expenditure).

Table 2 presents the prevalence rates of sensory loss and aid use by age and gender based on the full sample. Across the whole sample the prevalence of self-reported poor/fair vision for near or long distance was 80.2%. Only 10% of the sample wore glasses regularly. The prevalence of poor/fair self-rated vision for either long or near distance and long distance only was highest for the 70–74 year age group. The prevalence of DSL across the whole sample was 57.2%, increasing by age and was the highest for the 70–74 year age group (60.0%). This age group also had the highest prevalence for wearing glasses regularly. Across the whole sample the prevalence of self-rated poor/fair hearing was 64.9% on average, and was the highest in the 75 years and over age group as was this age groups' wearing of hearing aids. However, hearing aid use was extremely low across the whole sample (0.5–1.4%).

Men in the 70–74 year age range consistently had the highest prevalence of self-rated poor/fair short or long vision, poor/fair hearing and DSL although the respondents 75 years and over were the highest group to regularly use glasses or wear hearing aids. The pattern for women is different. Women in the 60–64 age group reported the highest prevalence of short or long poor/fair vision while women in the 70–74 age group reported the highest prevalence of wearing glasses regularly. For women, the 75 years and over age group reported the highest prevalence of poor/fair hearing and DSL. The youngest age group (those 60–64 years) had the highest prevalence for wearing hearing aids.

In summary the prevalence rates for short or long poor/fair vision, poor/fair hearing, and DSL are high in this older sample. However, it should be noted that the prevalence range across the age groups is small. Overall, in comparison to the prevalence of poor/fair vision, poor/fair hearing and DSL, only a small proportion used glasses (10.0% regularly and 20.1% from time to time) or wore hearing aid/s (0.8%). Glasses and hearing aid use were much lower than the proportion of respondent's reporting poor/fair vision (80.2%) or poor/fair hearing (64.9%) demonstrating an unmet need.

### Prevalence of Poor/fair Vision/hearing and Unmet Needs by Education, Rurality, and SES

Table 3 provides the sensory loss and unmet needs for sensory aids by SES. As is apparent from Table 3, there are significant differences by education and SES in terms of the prevalence of sensory loss, the use of sensory aids and unmet needs. Table 3 indicates that: (1) Respondents with primary or under schooling had the highest proportion of vision loss, hearing loss, and dual sensory loss as well as the highest proportion of unmet needs for glasses and hearing aid/s; respondents with college or above degrees had the highest proportion of wearing a hearing aid, wearing glasses regularly, and those with secondary schooling had the highest proportion of wearing glasses from time to time. (2) Respondents in rural areas had a higher proportion of vision loss, hearing loss, DSL and unmet needs for glasses and hearing aids, whilst respondents in the urban areas had a higher proportion of wearing glasses (either regularly or from time to time), and wearing a hearing aid; (3) Respondents with a worse living standard than the average had the highest proportion of vision loss, hearing loss, DSL and unmet needs for glasses and hearing aids whilst respondents with a better living standard had the highest proportion of wearing glasses regularly and wearing hearing aids, and those with an average living standard had the highest proportion of wearing glasses from time to time; (4) Respondents in the lowest two expenditure quintiles had the highest proportions of vision loss, hearing loss and DSL, and unmet needs for glasses and hearing aids whereas respondents in the highest two expenditure quintiles had the highest proportion of wearing glasses either regularly or from time to time.

### Relationships Between Vision Loss, Hearing Loss, DSL, Unmet Need, and Social Participation

Distribution (Table 4) and Spearman correlations (Table 5) were calculated between DSL, unmet needs and social activities, and a simple multivariate model (Table 6) controlling for age and gender was used to investigate the associations between self-rated poor/fair vision and hearing, unmet needs for sensory aids, and social participation. From Table 4 it was evident that older people with poor/fair vision and/or hearing, or unmet needs for glasses or hearing aids had significantly lower participation in all of the social activities, compared to those with excellent, very good or good vision or hearing.

The Spearman correlations shown in Table 5 further confirm that poor/fair vision and/or hearing, and the unmet needs for glasses or hearing aids are negatively associated with participation in all types of social activities.

Since vision and hearing capacities were strongly correlated with age, multivariate binominal logit or ordered logit models were conducted to examine the conditional associations between DSL and social activities by controlling for age group, gender, poor/fair vision/hearing, DSL, and the unmet needs for glasses or hearing aids into the regression models. The regression results are presented in Table 6.

From Table 6, it is evident that once other variables were controlled for, participation in social activity (the number of social activity types and activities of helping others) decreases significantly with age. This was however not the case for the social activities for leisure and learning. Furthermore, whilst the unmet needs for glasses was significantly related with all activity types, poor/fair vision or hearing and the unmet needs for hearing aids did not have significant influences on any of the social activities.

It must be noted however that the sample size of people wearing hearing aids was very small in this survey hence its related results need to be treated cautiously. In general, a Pseudo *R*-square value within 0.2 to 0.4 represents excellent fit. The Pseudo *R*-square in our final model is 0.01–0.04, less than the level of excellent fit, but we argue that it serves our purpose to investigate the conditional associations between social participation, vision/hearing loss, and the use of sensory aids. The low level of model fit could due to other factors, which are not included in this study, but have influence on later life social participation, such as severe disabilities and health conditions (mobility difficulty, blindness, deaf, psychological problems etc.), which were beyond the scope of this study.

## Discussion

Results of the current study are that self-assessed sensory loss [vision loss (defined as self-rated poor/fair vision), hearing loss (defined as self-rated poor/fair hearing), and DSL (defined as self-rated poor/fair vision and hearing)] are highly prevalent conditions experienced by older adults in China. Vision loss was more prevalent (80.2%) than hearing loss (64.9%) and DSL was experienced by over half of the cohort (57.2%).

The prevalence rates of sensory loss in the present study are higher in comparison to the prevalence of vision and hearing loss in older people in Western countries. For example, in Australia, the prevalence of vision loss in Australia ranges from 4.37 to 46.15% (28) for indigenous and non-indigenous people aged 60 years and over. The prevalence of hearing loss in Western countries ranges from 45.5 to 100% with large variations by age (29). The prevalence of DSL in the current study is also higher than in Western countries. In an Australian study (30), based on vision and audiometric assessments, the prevalence of DSL for those aged 60 years and over, was 6.93%. In comparison to the present study, a lower prevalence rate of DSL has also been reported by researchers in other countries such as the US [Brennan et al. (31) estimated the rate of DSL as 22.5%] and Japan [Harada et al. (32) estimated the prevalence range of DSL as 3% in cohorts aged 60–69 years to 21.9% in those aged 80 years and older].

Other population-based studies of vision and hearing loss in China found similar results to the present study. Based on 4 datasets collected in 2012 (National Rural Vision Care Survey, Private Optometrist Survey, County Hospital Eye Care, Rural School Vision Care Survey Survey), Bai et al. (22) found a similar prevalence rate (61%) of self-reported vision loss for people aged 50 and plus.

According to a survey of 6,984 older adults, Gong et al. (11) found the prevalence of hearing loss to be 58.9% for those aged 65 years and above. Based on the 2005–2006 cycle of the China National Health and Nutritional Examination Survey, Lin et al. (33) found the prevalence of hearing loss to be 63.1% for Chinese aged 70 years and over. These three rates are very close to the findings of the present study (80.2% for vision loss and 64.9% for hearing loss).

This differences in the prevalence of vision loss, hearing, and DSL between the current study to those conducted in Western countries is not surprising since there are country disparities in the general health status of populations, exposures to environmental and occupational hazards, identification of sensory loss (measures of visual acuity and audiometric data have often been used, whereas in the current study, self assessed sensory loss was used), access to healthcare and service delivery design for those with sensory losses. The Global Aging Watch Index (34) indicated that the health status of older people aged 60 years and older in China in 2014 was ranked 58th in the world, which is much lower than the health status of older people in developed countries (Australia, Japan and New Zealand were ranked within the top 10, and the US and UK were ranked within the top 30).

Comparison between the Household, Income, and Labor Dynamics in Australia (HILDA) Survey (35) and CHARLS 2013 data also show differences in self-rated health, chronic conditions and ADL limitations amongst older men aged 60–64 years. In the Australian dataset (HILDA), 25.5% of respondents reported poor/fair general health, 42.0% reported having any chronic diseases/conditions and 14.7% of respondents reported any ADL limitations. For the same age group in China (CHARLS data), the rates were 76.5, 73.1, and 11.3%, respectively. In comparison to Australians, Chinese older people are much more likely to report poor/fair general health and have chronic diseases, although they are slightly less limited in ADL limitations. Country differences in sensory loss prevalence as measured by self-report may also be influenced by cultural understandings of terms such as “fair” and “poor.” These linguistic nuances need to be further investigated.

Gender differences for the prevalence of poor/fair vision and hearing impairment in this study were evident but not large. Whilst there were slightly more women reporting vision loss than men, the opposite occurred for hearing loss. This is consistent with previous research that supports these gender differences. According to Courtright and Lewallen (36), more women than men are affected by visual impairment and blindness possibly due to risk factors (social and cultural differences), access to services (for example, reduced services for women) and life expectancy (in most cultures, women have a longer life expectancy than men). A gender disparity is also evident in relation to hearing loss confirming previous studies (37), particularly since significantly more men than women are exposed to hazardous occupational noise (38). However, in terms of aid use women appear to be more disadvantaged than men although sensory aid use overall was very low across the whole sample. More men used glasses regularly and hearing aids than women. Little literature is available regarding gender disparities in hearing aid use, although outcomes of a cross-sectional survey based on 4,979 adult males and 3,410 females in Switzerland suggested that women reported a higher prevalence of daily and regular hearing aid use than men (39). In comparison, no gender differences were found in older adults' hearing aid usage based on data from the National Health and Nutrition Examination Survey 2005–2006 and 2009–2010 (40).

Education, financial security and rurality impacted negatively on self-reported vision and hearing. Those people with less education or who lived rurally or had a poorer living standard or earned less, had a higher prevalence of self-reported poor/fair vision or hearing. This is not surprising since it is common for people who have financial constraints or live rurally to have less access to healthcare (especially specific services as supplied by allied health professionals such as Optometrists and Audiologists) as well as experience poorer health outcomes compared to their metropolitan counterparts (41). There is also a disparity between low and high-income countries accessing services such as visual examinations (42). In an analysis of a worldwide population-based dataset, Vela et al. (42) found that the number of people accessing visual examinations was 10% in low income countries and 37% in high income countries. Some of the factors associated with visual examinations included older age, female gender, more education, and urban residence.

There was a low uptake of glasses and hearing aids in this study although self-reported poor/fair vision were in comparison, large. This is not surprising and has been confirmed in previous research. According to Kuang et al. (25), in China, the awareness of eye care and vision improvement through the uptake of glasses or surgery (for example cataract surgery) is lower than expected. Barriers to widespread use of devices mentioned by these authors included the cost of spectacle frames and lenses, a decreased need due to older people not engaging in distance vision activities and the idea that vision loss is part of the natural aging process. Cataract surgery is also still low in China. In a retrospective cross-sectional study conducted in Shanghai, Zhu et al. (43) reported that although the cataract surgery rate increased from 2006 to 2009 by 26.94%, it is still low and less than the target suggested by the WHO. Yet, in a recent study of 87 Chinese older patients with low vision or blindness Ma, Zhang and Xu (44) suggested that reading glasses and visual aids were effective and an economical means of improving far and near visual acuity.

In the current study, although the prevalence of hearing loss was large, the use of hearing aids was small, leading to a high rate of unmet needs. This was especially so for those with low education, poorer SES and in those who lived rurally. Again, this is not surprising since in China, there is overall a smaller than expected uptake of hearing aids or assistive devices. In a review of the literature, Ji et al. (23) reported that although digital hearing aids are available in China, the number of people using hearing aids is small compared with the proportion of older people with hearing loss. Factors suggested as barriers include: a traditional attitudes toward hearing loss in older people; financial reasons; worries about unfamiliarity of hearing aids; and inability to manipulate hearing aids. Although bone anchored hearing aids, middle ear implants, and cochlear implants are also available in China, there is little data regarding their uptake by the older adult population.

Social participation is particularly important for general health and well-being and older people are encouraged to participate in social activities even if they have health difficulties (45). Social participation is impacted by sensory loss and associated communication difficulties (17). The results of this study suggest that people with poor/fair vision, poor/fair hearing or DSL, and high unmet needs for glasses or hearing aids, had significantly lower participation in social activities, compared to those with good, very good or excellent vision or hearing. This finding is especially important as there have been calls worldwide by peak bodies, consumer groups and governments to increase the participation of older people in civic life (46). Social participation is a key platform for active and healthy aging concepts. The reason for this policy approach has often been economic, we need more older people to be economically productive, however civic participation is a basic human right for all ages (47). Disabilities associated with vision and hearing loss in older people are often considered a “normal” part of aging by both older people themselves and health professionals. However, there is strong evidence that appropriate sensory screening and rehabilitation programs can greatly improve the quality of life for older people. Sensory loss in older people is often undetected and underestimated. Hearing loss in older adults for example is a leading cause of adult hearing handicap in the US and is originally slow to develop however, if left untreated impacts significantly on the person and significant others (48). Awareness of sensory loss, improved education and screening leads to early identification, and management such as fitting of aids and communication training, leading to improved well-being and quality of life.

## Conclusions

In this paper we have provided strong evidence that the prevalence of sensory loss is a significant health issue for older Chinese that impacts on their ability to engage in social and work activities. However, unmet needs for sensory aids are high in China.

Regular vision and hearing screening for older Chinese is recommended and increasing the uptake of sensory aids is an important goal to reduce disability in those with sensory loss. The health system in China predominantly relies on doctors and nurses to deliver health care, mainly in tertiary hospital settings (49). The (2017–2025) Chinese State Council plan for the prevention and treatment of chronic diseases emphasizes better co-ordination of prevention and treatment and improved primary health care services (50). While the primary health care reforms seek to increase the numbers of doctors and nurses working in primary care settings, training in sensory loss screening, and rehabilitation and communication approaches is limited. The primary health care setting provides an important opportunity to meet the needs of older people with sensory loss through screening and encouragement in the use of aids.

The availability of audiologists or doctors and nurses with audiology training is low in China and thus identification of hearing loss and access to audiological services is uncommon especially for older people with low financial resources. To increase the use of sensory aids in older people with sensory loss, reduced financial barriers to both screening and the cost of aids is needed.

We recommend that further training in sensory loss and rehabilitation be made available to primary care health professionals, particularly those who have high older patient caseloads. There is also a need for the training of hearing specialists in hearing care. Some multinational private hearing care companies are now setting up hearing training centers in China but public facilities are limited (51). Finally, we recommend that consideration be given to the inclusion of greater screening and sensory aid reimbursements in medical insurance schemes in China.

## Study Limitations

CHARLS is an excellent longitudinal survey on China's health but our findings should be interpreted with some caution as there are limitations which should be noted. Firstly, we have only examined the cross-sectional associations between sensory loss and social participation without aiming to understand the underlying causality. Secondly, CHARLS is a representative community sample in urban and rural areas but it does not cover the ~1% older people living in an institution. Thirdly, CHARLS uses self-reported measures of sensory loss which has great ecological validity and reflective of sensory disability, yet might be subject to reporting bias and prone to cultural difference in conceptualisations of sensory loss, thus potentially limiting comparisons of our findings with similar studies in western developed countries. Future research could further explore the casual relationships between DSL and social participation and well-being using longitudinal data when more waves of CHARLS survey data become available.

## Ethics Statement

The original CHARLS survey data was approved by the Ethical Review Committee of Peking University, and all participants signed informed consent at the time of participation. There is no need for ethics approval for second hand data users.

## Author Contributions

CH and CB conceptualized the paper. CG conducted all the statistical analyses. CH drafted the paper and CG and CB revised the paper. All authors have final approval of the published article and agree to be accountable for all aspects of the work in ensuring that questions related to the accuracy or integrity of any part of the work are appropriately investigated and resolved.

### Conflict of Interest Statement

The authors declare that the research was conducted in the absence of any commercial or financial relationships that could be construed as a potential conflict of interest.

## References

[1] World Health Organization Vision Impairment and Blindness. Fact sheet (2017). Available online at: http://www.who.int/mediacentre/factsheets/fs282/en/ (Accessed June 2018).

[2] World Health Organization WHO Global Estimates on Prevalence of Hearing Loss. Mortality and Burden of Diseases and Prevention of Blindness and Deafness (2012). Available online at: http://www.who.int/pbd/deafness/WHO_GE_HL.pdf (Accessed June 2018).

[3] World Health Organization Estimates (2018). Available online at: http://www.who.int/deafness/estimates/en/ (Accessed June 2018).

[4] Olusanya BO Neumann KJ Saunders JE Policy & practice. The global burden of disabling hearing impairment: a call to action. Bull World Health Organ. (2014) 92:367–73. 10.2471/BLT.13.128728 24839326 PMC4007124

[5] Heine C Dual sensory loss. In: Pancha NA, editor. Encyclopaedia of Geropsychology. Singapore: Springer (2016). p. 1–6.

[6] Zhao J Ellwein LB Cui H Ge J Guan H Lv J. Prevalence of vision impairment in older adults in rural China. Ophthalmology (2010) 117:409–16. 10.1016/j.ophtha.2009.11.023 20079923 PMC6029941

[7] Zhang G Li Y Teng X Wu Q Gong H Ren F. Prevalence and causes of low vision and blindness in Baotou: a cross-sectional study. Medicine (2016) 95:e4905. 10.1097/MD.0000000000004905 27631267 PMC5402610

[8] Wang B Congdon N Bourne R Li Y Cao K Zhao A. Burden of vision loss associated with eye disease in China 1990-2020: findings from the Global Burden of Disease Study 2015. Br J Ophthalmol. (2018) 102:220–4. 10.1136/bjophthalmol-2017-310333 28607177

[9] Yu ESH Kean YM Slymen DJ Liu WT Zhang M Katzman R Self-perceived health and 5-year mortality risk among the elderly in Shanghai, China. Am J Epidemiol. (1998) 147:880–90. 10.1093/oxfordjournals.aje.a009542 9583719

[10] Liu XZ Xu LR Hu Y Nance WE Sismanis A Zhang L. Epidemiological studies on hearing impairment with reference to genetic factors in Sichuan, China. Ann Otol Rhinol Laryngol. (2001) 110:356–63. 10.1177/000348940111000412 11307913

[11] Gong R Hu X Gong C Long M Han R Zhou L. Hearing loss prevalence and risk factors among older adults in China. Int J Audiol. (2018) 57:354–9. 10.1080/14992027.2017.1423404 29400111

[12] Heine C Browning C. Dual sensory loss in older adults: a systematic review. Gerontologist (2015) 55:913–28. 10.1093/geront/gnv074 26315316

[13] Swenor BK Ramulu PY Willis JR Friedman D Lin FR. The prevalence of concurrent hearing and vision impairment in the United States. JAMA Intern Med. (2013) 173:312–3. 10.1001/jamainternmed.2013.1880 23338042 PMC3875214

[14] Caban AJ Lee DJ Gomez-Marin O Lam BL Zheng D. Prevalence of concurrent hearing and visual impairment in US adults: the national health interview survey, 1997-2002. Am J Public Health (2005) 95:1940–2. 10.2105/AJPH.2004.056671 16195516 PMC1449463

[15] Chou KL Chi I. Combined effect of vision and hearing impairment on depression in elderly Chinese. [Research Support, non-U.S. Gov't]. Int J Geriatr Psychiatry (2004) 19:825–32. 10.1002/gps.1174 15352139

[16] Anderson Rooth M The prevalence and impact of vision and hearing loss in the elderly. North Carol Med J. (2017) 78:118–20. 10.18043/ncm.78.2.118 28420775

[17] Heine C Browning C. Communication and psychosocial consequences of sensory loss in older adults: overview and rehabilitation directions. Disabil Rehabil. (2002) 24:763–73. 10.1080/09638280210129162 12437862

[18] Gopinath B Schneider J McMahon CM Burlutsky G Leeder SR Mitchell P. Dual sensory impairment in older adults increases the risk of mortality: a population-based study. PLoS ONE (2013) 8:e55054. 10.1371/journal.pone.0055054 23469161 PMC3587637

[19] Heine C Browning C Communication and psychosocial profiles of older adults with sensory loss - a focus group study. Aging Soc. (2004) 24:113–30. 10.1017/S0144686X03001491

[20] Knudsen LV Öberg M Nielsen C Naylor G Kramer SE. Factors influencing help seeking, hearing aid uptake, hearing aid use and satisfaction with hearing aids: a review of the literature. Trends Amplif. (2010) 14:127–54. 10.1177/1084713810385712 21109549 PMC4111466

[21] Li L Lam J Lu Y Ye Y Lam DS Gao Y. Attitudes of students, parents, and teachers toward glasses use in rural China. Arch Opthalmol. (2010) 128:759–65. 10.1001/archophthalmol.2010.73 20547954

[22] Bai Y Yi H Zhang L Shi Y Ma X Congdon N Vision in Rural China and the Failure of the Vision Care System. Rural Education Action Project. Working paper 259. (2013). Available online at: www.reapchina.org/reap.stanford.edu (Accessed June 2018).

[23] Ji F Chen AT Wang QJ Hearing loss in the aged: status and interventions in China, hearing, balance and communication (2015) 13:51–7. 10.3109/21695717.2015.1032719

[24] Zhoa Y Hu Y Smith JP Strauss J Yang G Cohort profile: the China health and retirement longitudinal study (CHARLS). Int J Epidemiol. (2014) 43:61–8. 10.1093/ije/dys203 23243115 PMC3937970

[25] Kuang TM Tsai SY Liu CJL Ko YC Lee SM Chou P. Seven-year incidence of uncorrected refractive error among an elderly Chinese population in Shihpai, Taiwan: The Shihpai Eye Study. Eye (2016) 30:570–6. 10.1038/eye.2015.276 26795416 PMC5108537

[26] Gong CH Kendig H He X. Factors predicting health services use among older people in China: an analysis of the China health and retirement longitudinal study 2013. BMC Health Services Res. (2016) 16:63. 10.1186/s12913-016-1307-8 26892677 PMC4758158

[27] National, Bureau of Statistics of China (NBS). Available online at: http://www.stats.gov.cn/english/

[28] Foreman J Keel S Xie J Van, Wijngaarden P Crowston J Taylor HR The National Eye Health Survey 2016. (2016). Available online at: http://www.vision2020australia.org.au/uploads/resource/250/National-Eye-Health-Survey_Full-Report_FINAL.pdf (Accessed June 2018).

[29] Cruikshanks KJ Nondahl DM Tweed TS Wiley TL Klein BEK Klein R Education, occupation, noise exposure history and the 10-yr cumulative incidence of hearing impairment in older adults. Hear Res. (2010) 264:3–9. 10.1016/j.heares.2009.10.008 19853647 PMC2868082

[30] Schneider J Gopinath B McMahon C Teber E Leeder SR Jin Wang J. Prevalence and 5-year incidence of dual sensory impairment in an older Australian population. AEP (2012) 22:295–301. 10.1016/j.annepidem.2012.02.004 22382082

[31] Brennan M Su YP Horowitz A. Longitudinal associations between dual sensory impairment and everyday competence among older adults. J Rehabil Res Dev. (2006) 43:777–92. 10.1682/JRRD.2005.06.0109 17310427

[32] Harada S Nishiwaki Y Michikawa T Kikuchi Y Iwasawa S Nakano M Gender difference in the relation- ships between vision and hearing impairments and negative well-being. Prev Med. (2008) 47:433–7. 10.1016/j.ypmed.2008.06.011 18619483

[33] Lin FR Thorpe R Gordon-Salant S Ferrucci L. Hearing loss prevalence and risk factors among older adults in the United States. J Gerontol. (2011) 66A:582–90. 10.1093/gerona/glr002 21357188 PMC3074958

[34] Global Age Watch Index Global Age Watch Index 2014:Insight Report. HelpAge International (2014). Available online at: https://cdn.24.co.za/files/Cms/General/d/689/952b033f6fa2490aa4f2b4c605e7f039.pdf (Accessed June 2018).

[35] The Household Income and Labour Dynamics in Australia (HILDA) Survey. (2018). Available online at: https://melbourneinstitute.unimelb.edu.au/hilda. (Accessed July 2018).

[36] Courtright P Lewallen S. Why are we addressing gender issues in vision loss? Commun Eye Health (2009) 22:17–9. 19888362 PMC2760274

[37] Heine C Browning C Cowlishaw S Kendig H. Trajectories of older adults' hearing difficulties: examining the influence of health behaviors and social activity over 10 years. Geriatr Gerontol Int. (2013) 13:911–8. 10.1111/ggi.12030 23311873

[38] Feder K Michaud D McNamee J Fitzpatrick E Davies H Leroux T. Prevalence of hazardous occupational noise exposure, hearing loss, and hearing protection usage among a representative sample of working Canadians. J Occupat Environ Med. (2017) 59:92–113. 10.1097/JOM.0000000000000920 28045804 PMC5704673

[39] Staehelin K Bertoli S Probst R Schindler C Dratva J Stutz EZ Gender and hearing aids: patterns of use and determinants of non-regular use. Ear Hear. (2011) 32:e26–37. 10.1097/AUD.0b013e3182291f94 21795978

[40] Bainbridge KE Ramachandran V. Hearing aid use among older U.S. adults; the National Health and Nutrition Examination Survey, 2005-2006 and 2009-2010. Ear Hear. (2014) 35:289–94. 10.1097/01.aud.0000441036.40169.29 24521924 PMC3999213

[41] Thomas SL Wakerman J Humphreys JS. Ensuring equity of access to primary health care in rural and remote Australia - what core services should be locally available? Int J Equity Health (2015) 14:111. 10.1186/s12939-015-0228-1 26510998 PMC4625941

[42] Vela C Samson E Zunzunegui MV Haddad S Aubin MJ Freeman EE. Eye care utilization by older adults in low, middle, and high income countries. BMC Ophthalmol. (2012) 12:5. 10.1186/1471-2415-12-5 22471351 PMC3378437

[43] Zhu M Zhu J Lu L He X Zhao R Zou H. Four-year analysis of cataract surgery rates in Shanghai, China: a retrospective cross-sectional study. BMC Opthalmol. (2014) 14:3. 10.1186/1471-2415-14-3 24410915 PMC3893501

[44] Ma JX Zhang L Xu NN Cause of low vision and blind in elderly and the application of optical aids in their rehabilitation. Guoji Yanke Zazhi (2017) 17:1599–601. 10.1590/S0041-87812004000400001

[45] Galenkamp H Deeg DJH. Increasing social participation of older people: are there different barriers for those in poor health? Introduction to the special section. Eur J Aging (2016) 13:87–90. 10.1007/s10433-016-0379-y 28804373 PMC5550606

[46] World Health Organization World report on ageing and health 2015. Geneva: WHO (2015). Available online at: http://www.who.int/ageing/events/world-report-2015-launch/en/ (Accessed June 2018).

[47] United, Nations Human Rights Office of the High Commissioner (nd),. Human Rights for Older People. Available online at: https://www.ohchr.org/EN/Issues/OlderPersons/Pages/OlderPersonsIndex.aspx (Accessed, June 2018).

[48] Li-Korotsky HS Age-related hearing loss: quality of care for quality of life. Gerontologist (2012) 52:265–71. 10.1093/geront/gnr159 22383543

[49] Chen Z. Launch of the health-care reform plan in China. Lancet (2009) 373:1322–4. 10.1016/S0140-6736(09)60753-4 19376436

[50] Kong L-Z. China's Medium-to-Long Term Plan for the Prevention and Treatment of Chronic Diseases (2017-2025) under the Healthy China Initiative. Chronic Dis Transl Med. (2017) 3:135–7. 10.1016/j.cdtm.2017.06.004 29063067 PMC5643768

[51] Sonova Opens Audiology Training Center in Suzhou China The Hearing Review (2017). Available online at: http://www.hearingreview.com/2017/05/sonova-opens-audiology-training-center-suzhou-china/ (Accessed November 2018).

